# Bis(perchlorato-κ*O*)tetra­kis[1-(2-pyridyl)-4-(4-pyridylmethyl-κ*N*)piperazine]cadmium(II)

**DOI:** 10.1107/S1600536809004437

**Published:** 2009-02-13

**Authors:** Gregory A. Farnum, Robert L. LaDuca

**Affiliations:** a Lyman Briggs College, Department of Chemistry, Michigan State University, East Lansing, MI 48825, USA

## Abstract

In the title compound, [Cd(ClO_4_)_2_(C_15_H_18_N_4_)_4_], the Cd^II^ ion is coordinated in a slightly distorted octa­hedral environment by two *trans* monodentate perchlorate ligands and four 1-(2-pyrid­yl)-4-(4-pyridylmeth­yl)piperazine (pmpp) ligands. In the crystal structure, mol­ecules are organized into layers parallel to the *ab* plane by C—H⋯O inter­actions. Similar inter­actions promote the stacking of these layers into the three-dimensional crystal structure.

## Related literature

For a silver nitrate supra­molecular complex and the synthesis of pmpp, see: Farnum *et al.* (2009 ▶).
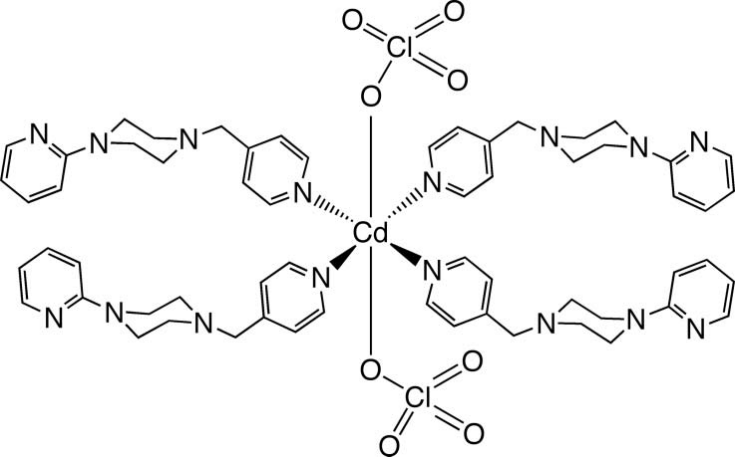


## Experimental

### 

#### Crystal data

[Cd(ClO_4_)_2_(C_15_H_18_N_4_)_4_]
*M* *_r_* = 1328.64
Monoclinic, 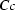

*a* = 34.958 (4) Å
*b* = 9.1736 (11) Å
*c* = 24.153 (3) Å
β = 126.8740 (10)°
*V* = 6196.4 (13) Å^3^
*Z* = 4
Mo *K*α radiation
μ = 0.51 mm^−1^
*T* = 173 K
0.20 × 0.16 × 0.12 mm

#### Data collection

Bruker SMART 1K diffractometer
Absorption correction: multi-scan (*SADABS*; Sheldrick, 2007 ▶) *T*_min_ = 0.875, *T*_max_ = 0.941
34115 measured reflections
13967 independent reflections
11273 reflections with *I* > 2σ(*I*)
*R*_int_ = 0.052

#### Refinement

*R*[*F* ^2^ > 2σ(*F* ^2^)] = 0.041
*wR*(*F* ^2^) = 0.084
*S* = 0.98
13967 reflections
784 parameters
2 restraints
H-atom parameters constrained
Δρ_max_ = 0.70 e Å^−3^
Δρ_min_ = −0.41 e Å^−3^
Absolute structure: Flack (1983 ▶), with 6755 Friedel pairs
Flack parameter: −0.041 (13)

### 

Data collection: *SMART* (Bruker, 2006 ▶); cell refinement: *SAINT-Plus* (Bruker, 2006 ▶); data reduction: *SAINT-Plus*; program(s) used to solve structure: *SHELXS97* (Sheldrick, 2008 ▶); program(s) used to refine structure: *SHELXL97* (Sheldrick, 2008 ▶); molecular graphics: *CrystalMaker* (Palmer, 2007 ▶); software used to prepare material for publication: *SHELXL97*.

## Supplementary Material

Crystal structure: contains datablocks I, global. DOI: 10.1107/S1600536809004437/lh2771sup1.cif

Structure factors: contains datablocks I. DOI: 10.1107/S1600536809004437/lh2771Isup2.hkl

Additional supplementary materials:  crystallographic information; 3D view; checkCIF report

## Figures and Tables

**Table 1:** Hydrogen-bond geometry (Å, °)

*D*—H⋯*A*	*D*—H	H⋯*A*	*D*⋯*A*	*D*—H⋯*A*
C29—H29⋯O1^i^	0.95	2.54	3.406 (6)	152
C54—H54*A*⋯O7^ii^	0.99	2.57	3.348 (5)	135

Symmetry codes: (i) 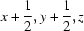; (ii) 
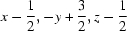
.

**Fig. 1.**
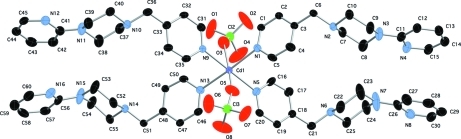
The molecular structure of the title compound. Color codes: violet Cd, green Cl, light blue N, red O, black C. H-atom positions are shown as grey sticks.

**Fig. 2.**
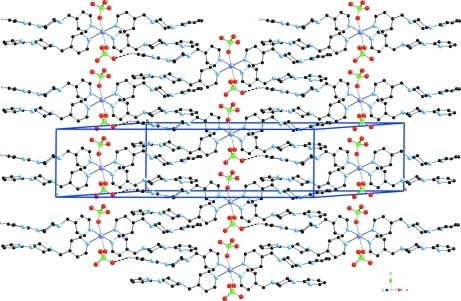
A supramolecular layer formed by C—H···O interactions between molecules of the title compound.

**Fig. 3.**
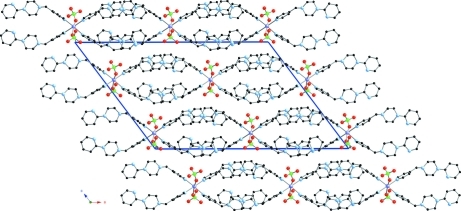
Part of the crystal structure showing the AB stacking pattern of supramolecular layers.

## supplementary crystallographic information

### Comment

In an attempt to prepare a cadmium perchlorate coordination polymer containing pmpp (Farnum *et al.*, 2009), colourless blocks of the title compound were obtained. The asymmetric unit contains a single molecule, [Cd(pmpp)_4_(ClO_4_)_2_], wherein an octahedrally coordinated Cd^II^ ion is bound by two *trans* monodentate perchlorato ligands and four pmpp ligands, bound *via* N atoms within their 4-pyridyl termini (Fig. 1). The title compound crystallizes in the noncentrosymmetric monoclinic space group *Cc* without appreciable racemic twinning, as evidenced by a Flack parameter (Flack, 1983) of -0.043 (13).

Individual [Cd(pmpp)_4_(ClO_4_)_2_] molecules aggregate through C—H···O interactions, constructing *pseudo* layers coincident with the *ab* crystal planes (Fig. 2). In turn these layers stack in an AB pattern along the *c* crystal direction by a different C—H···O pathway (Fig. 3).

### Experimental

All chemicals were obtained commercially. Cadmium perchlorate hexahydrate (20 mg, 0.064 mmol) was dissolved in 1.5 ml water in a glass vial at 298 K. A 0.75 ml aliquot of a 1:1 water–ethanol mixture was carefully layered onto the aqueous solution, followed by 1.5 ml of an ethanolic solution of pmpp (32.5 mg, 0.128 mmol). Colourless blocks of the title compound formed after 2 weeks.

### Refinement

All H atoms bound to C atoms were placed in calculated positions, with C—H = 0.95 Å or 0.99 Å, and refined in riding mode with *U*_iso_ = 1.2*U*_eq_(C).

### Crystal data

**Table:**

[Cd(ClO_4_)_2_(C_15_H_18_N_4_)_4_]	*F*(000) = 2760
*M**_r_* = 1328.64	*D*_x_ = 1.424 Mg m^−^^3^
Monoclinic, *C**c*	Mo *K*α radiation, λ = 0.71073 Å
Hall symbol: C-2yc	Cell parameters from 34115 reflections
*a* = 34.958 (4) Å	θ = 1.5–28.3°
*b* = 9.1736 (11) Å	µ = 0.51 mm^−^^1^
*c* = 24.153 (3) Å	*T* = 173 K
β = 126.874 (1)°	Block, colourless
*V* = 6196.4 (13) Å^3^	0.20 × 0.16 × 0.12 mm
*Z* = 4	

### Data collection

**Table:**

Bruker SMART 1K diffractometer	13967 independent reflections
Radiation source: fine-focus sealed tube	11273 reflections with *I* > 2σ(*I*)
graphite	*R*_int_ = 0.052
ω scans	θ_max_ = 28.3°, θ_min_ = 1.5°
Absorption correction: multi-scan (*SADABS*; Sheldrick, 2007)	*h* = −44→44
*T*_min_ = 0.875, *T*_max_ = 0.941	*k* = −12→12
34115 measured reflections	*l* = −30→30

### Refinement

**Table:**

Refinement on *F*^2^	Secondary atom site location: difference Fourier map
Least-squares matrix: full	Hydrogen site location: inferred from neighbouring sites
*R*[*F*^2^ > 2σ(*F*^2^)] = 0.041	H-atom parameters constrained
*wR*(*F*^2^) = 0.084	*w* = 1/[σ^2^(*F*_o_^2^) + (0.0388*P*)^2^] where *P* = (*F*_o_^2^ + 2*F*_c_^2^)/3
*S* = 0.98	(Δ/σ)_max_ = 0.001
13967 reflections	Δρ_max_ = 0.70 e Å^−^^3^
784 parameters	Δρ_min_ = −0.41 e Å^−^^3^
2 restraints	Absolute structure: Flack (1983), with 6755 Friedel pairs
Primary atom site location: structure-invariant direct methods	Flack parameter: −0.041 (13)

### Special details

**Table:**

Geometry. All e.s.d.'s (except the e.s.d. in the dihedral angle between two l.s. planes) are estimated using the full covariance matrix. The cell e.s.d.'s are taken into account individually in the estimation of e.s.d.'s in distances, angles and torsion angles; correlations between e.s.d.'s in cell parameters are only used when they are defined by crystal symmetry. An approximate (isotropic) treatment of cell e.s.d.'s is used for estimating e.s.d.'s involving l.s. planes.
Refinement. Refinement of *F*^2^ against ALL reflections. The weighted *R*-factor *wR* and goodness of fit *S* are based on *F*^2^, conventional *R*-factors *R* are based on *F*, with *F* set to zero for negative *F*^2^. The threshold expression of *F*^2^ > σ(*F*^2^) is used only for calculating *R*-factors(gt) *etc*. and is not relevant to the choice of reflections for refinement. *R*-factors based on *F*^2^ are statistically about twice as large as those based on *F*, and *R*- factors based on ALL data will be even larger.

### Fractional atomic coordinates and isotropic or equivalent isotropic displacement parameters (Å^2^)

**Table:**

	*x*	*y*	*z*	*U*_iso_*/*U*_eq_	
Cd1	0.944502 (8)	0.40038 (2)	0.851212 (10)	0.02197 (6)	
Cl2	0.89524 (3)	0.08947 (10)	0.73405 (4)	0.0314 (2)	
Cl3	0.98569 (3)	0.74096 (9)	0.95355 (4)	0.0313 (2)	
O1	0.85179 (12)	0.0194 (5)	0.70850 (18)	0.0832 (13)	
O2	0.93073 (14)	−0.0123 (5)	0.7526 (2)	0.1008 (15)	
O3	0.91053 (10)	0.1719 (3)	0.79483 (15)	0.0531 (8)	
O4	0.88951 (14)	0.1820 (5)	0.68378 (18)	0.1070 (17)	
O5	0.98435 (12)	0.5974 (3)	0.93104 (17)	0.0626 (9)	
O6	0.97222 (13)	0.7354 (4)	0.99851 (18)	0.0806 (12)	
O7	1.03264 (12)	0.7964 (4)	0.99005 (18)	0.0760 (11)	
O8	0.95294 (14)	0.8286 (4)	0.89449 (17)	0.0869 (13)	
N1	1.01789 (10)	0.2833 (3)	0.90920 (15)	0.0264 (7)	
N2	1.19180 (9)	0.1926 (3)	1.00871 (14)	0.0238 (6)	
N3	1.26147 (11)	0.2236 (4)	0.98396 (16)	0.0420 (9)	
N4	1.27668 (11)	0.2627 (4)	0.90449 (17)	0.0383 (8)	
N5	0.96399 (10)	0.5031 (3)	0.78326 (15)	0.0291 (7)	
N6	1.04588 (10)	0.5644 (3)	0.66929 (15)	0.0271 (7)	
N7	1.13255 (12)	0.5004 (6)	0.68796 (18)	0.0714 (14)	
N8	1.17255 (12)	0.5530 (3)	0.64101 (17)	0.0386 (8)	
N9	0.92928 (10)	0.3042 (3)	0.92556 (14)	0.0250 (6)	
N10	0.83893 (10)	0.2041 (3)	1.02504 (14)	0.0273 (7)	
N11	0.74481 (11)	0.2154 (4)	0.98869 (15)	0.0398 (8)	
N12	0.69473 (11)	0.1813 (3)	1.02141 (15)	0.0331 (7)	
N13	0.86879 (10)	0.5004 (3)	0.78932 (14)	0.0244 (6)	
N14	0.69229 (9)	0.5659 (3)	0.68151 (14)	0.0230 (6)	
N15	0.61776 (11)	0.4882 (5)	0.69084 (16)	0.0579 (11)	
N16	0.59698 (12)	0.5186 (4)	0.76485 (17)	0.0407 (8)	
C1	1.02538 (12)	0.1405 (4)	0.91269 (19)	0.0272 (8)	
H1	0.9985	0.0774	0.8916	0.033*	
C2	1.07003 (12)	0.0789 (4)	0.94527 (19)	0.0281 (8)	
H2	1.0733	−0.0241	0.9463	0.034*	
C3	1.11017 (12)	0.1672 (4)	0.97649 (17)	0.0242 (8)	
C4	1.10261 (13)	0.3154 (4)	0.9727 (2)	0.0397 (10)	
H4	1.1289	0.3807	0.9928	0.048*	
C5	1.05745 (16)	0.3683 (4)	0.9400 (3)	0.0404 (12)	
H5	1.0534	0.4710	0.9388	0.049*	
C6	1.15967 (14)	0.1048 (4)	1.0152 (2)	0.0284 (9)	
H6A	1.1734	0.0965	1.0648	0.034*	
H6B	1.1576	0.0054	0.9976	0.034*	
C7	1.17845 (12)	0.1844 (5)	0.93855 (19)	0.0332 (9)	
H7A	1.1454	0.2213	0.9051	0.040*	
H7B	1.1792	0.0815	0.9269	0.040*	
C8	1.21230 (13)	0.2733 (5)	0.9328 (2)	0.0427 (10)	
H8A	1.2036	0.2631	0.8857	0.051*	
H8B	1.2097	0.3776	0.9407	0.051*	
C9	1.27530 (13)	0.2287 (5)	1.0534 (2)	0.0411 (10)	
H9A	1.2756	0.3312	1.0666	0.049*	
H9B	1.3080	0.1888	1.0860	0.049*	
C10	1.24062 (12)	0.1407 (4)	1.05854 (19)	0.0333 (9)	
H10A	1.2425	0.0366	1.0496	0.040*	
H10B	1.2497	0.1492	1.1059	0.040*	
C11	1.29431 (13)	0.2327 (4)	0.96979 (19)	0.0324 (9)	
C12	1.34343 (13)	0.2060 (4)	1.0214 (2)	0.0336 (9)	
H12	1.3556	0.1870	1.0680	0.040*	
C13	1.37318 (14)	0.2085 (4)	1.0014 (2)	0.0389 (10)	
H13	1.4063	0.1891	1.0346	0.047*	
C14	1.35531 (15)	0.2388 (4)	0.9344 (2)	0.0394 (10)	
H14	1.3756	0.2423	0.9205	0.047*	
C15	1.30730 (15)	0.2637 (4)	0.8881 (2)	0.0390 (10)	
H15	1.2947	0.2830	0.8414	0.047*	
C16	0.97696 (19)	0.4129 (4)	0.7541 (3)	0.0389 (12)	
H16	0.9755	0.3111	0.7597	0.047*	
C17	0.99227 (15)	0.4589 (4)	0.7163 (2)	0.0399 (10)	
H17	1.0012	0.3895	0.6968	0.048*	
C18	0.99468 (12)	0.6056 (4)	0.70676 (19)	0.0290 (8)	
C19	0.98089 (12)	0.6997 (4)	0.73640 (19)	0.0319 (8)	
H19	0.9816	0.8019	0.7309	0.038*	
C20	0.96608 (13)	0.6461 (4)	0.77395 (19)	0.0299 (8)	
H20	0.9570	0.7133	0.7941	0.036*	
C21	1.01122 (16)	0.6611 (4)	0.6655 (2)	0.0376 (10)	
H21A	0.9832	0.6721	0.6164	0.045*	
H21B	1.0258	0.7586	0.6829	0.045*	
C22	1.09188 (13)	0.5742 (5)	0.73759 (19)	0.0397 (10)	
H22A	1.0878	0.5493	0.7736	0.048*	
H22B	1.1042	0.6751	0.7459	0.048*	
C23	1.12757 (15)	0.4696 (6)	0.7421 (2)	0.0624 (15)	
H23A	1.1590	0.4796	0.7878	0.075*	
H23B	1.1165	0.3680	0.7375	0.075*	
C24	1.08643 (14)	0.4962 (6)	0.6188 (2)	0.0492 (12)	
H24A	1.0733	0.3960	0.6086	0.059*	
H24B	1.0911	0.5236	0.5836	0.059*	
C25	1.05150 (13)	0.6004 (4)	0.61546 (18)	0.0335 (8)	
H25A	1.0634	0.7016	0.6220	0.040*	
H25B	1.0201	0.5939	0.5694	0.040*	
C26	1.17465 (13)	0.5306 (4)	0.6982 (2)	0.0368 (9)	
C27	1.21847 (15)	0.5353 (5)	0.7642 (2)	0.0427 (10)	
H27	1.2198	0.5196	0.8042	0.051*	
C28	1.21344 (16)	0.5778 (5)	0.6498 (2)	0.0397 (10)	
H28	1.2119	0.5926	0.6096	0.048*	
C29	1.25750 (16)	0.5836 (4)	0.7125 (2)	0.0404 (10)	
H29	1.2856	0.6011	0.7158	0.049*	
C30	1.25938 (15)	0.5628 (4)	0.7704 (2)	0.0436 (11)	
H30	1.2892	0.5676	0.8150	0.052*	
C31	0.93184 (14)	0.1620 (4)	0.9388 (2)	0.0361 (9)	
H31	0.9449	0.0992	0.9227	0.043*	
C32	0.91637 (13)	0.1013 (4)	0.9746 (2)	0.0345 (9)	
H32	0.9193	−0.0006	0.9832	0.041*	
C33	0.89661 (13)	0.1890 (4)	0.99785 (19)	0.0296 (8)	
C34	0.89430 (14)	0.3378 (4)	0.98476 (19)	0.0324 (9)	
H34	0.8815	0.4031	1.0003	0.039*	
C35	0.91075 (17)	0.3890 (4)	0.9490 (2)	0.0322 (11)	
H35	0.9088	0.4908	0.9405	0.039*	
C36	0.87977 (18)	0.1253 (5)	1.0372 (3)	0.0372 (12)	
H36A	0.8707	0.0222	1.0234	0.045*	
H36B	0.9065	0.1273	1.0873	0.045*	
C37	0.79610 (13)	0.1850 (5)	0.95317 (19)	0.0377 (10)	
H37A	0.7881	0.0801	0.9437	0.045*	
H37B	0.8027	0.2209	0.9210	0.045*	
C38	0.75448 (14)	0.2670 (5)	0.94099 (19)	0.0450 (11)	
H38A	0.7618	0.3726	0.9482	0.054*	
H38B	0.7259	0.2523	0.8927	0.054*	
C39	0.78663 (13)	0.2243 (5)	1.06098 (18)	0.0360 (9)	
H39A	0.7793	0.1786	1.0908	0.043*	
H39B	0.7948	0.3278	1.0747	0.043*	
C40	0.82859 (13)	0.1474 (4)	1.07100 (19)	0.0318 (9)	
H40A	0.8572	0.1594	1.1196	0.038*	
H40B	0.8215	0.0419	1.0622	0.038*	
C41	0.69918 (13)	0.2184 (4)	0.97106 (18)	0.0319 (8)	
C42	0.65933 (14)	0.2532 (4)	0.90413 (19)	0.0353 (9)	
H42	0.6630	0.2824	0.8698	0.042*	
C43	0.65010 (15)	0.1735 (4)	1.0039 (2)	0.0326 (10)	
H43	0.6465	0.1446	1.0384	0.039*	
C44	0.60962 (13)	0.2053 (4)	0.9389 (2)	0.0357 (9)	
H44	0.5789	0.2002	0.9288	0.043*	
C45	0.61478 (14)	0.2443 (4)	0.8891 (2)	0.0399 (10)	
H45	0.5872	0.2655	0.8436	0.048*	
C46	0.85807 (13)	0.6415 (4)	0.77122 (19)	0.0296 (8)	
H46	0.8830	0.7060	0.7823	0.035*	
C47	0.81227 (12)	0.6972 (4)	0.73715 (18)	0.0270 (8)	
H47	0.8064	0.7977	0.7254	0.032*	
C48	0.77520 (11)	0.6066 (4)	0.72029 (16)	0.0232 (7)	
C49	0.78600 (11)	0.4604 (4)	0.73832 (17)	0.0241 (7)	
H49	0.7616	0.3933	0.7273	0.029*	
C50	0.83285 (15)	0.4139 (4)	0.7726 (2)	0.0270 (9)	
H50	0.8397	0.3139	0.7849	0.032*	
C51	0.72491 (12)	0.6655 (4)	0.6807 (2)	0.0257 (9)	
H51A	0.7256	0.7600	0.7012	0.031*	
H51B	0.7125	0.6835	0.6322	0.031*	
C52	0.69999 (12)	0.5694 (4)	0.74789 (18)	0.0335 (9)	
H52A	0.6937	0.6690	0.7565	0.040*	
H52B	0.7337	0.5450	0.7854	0.040*	
C53	0.66746 (13)	0.4623 (5)	0.7488 (2)	0.0479 (12)	
H53A	0.6765	0.3616	0.7461	0.057*	
H53B	0.6714	0.4722	0.7927	0.057*	
C54	0.61010 (13)	0.4890 (5)	0.62463 (19)	0.0429 (11)	
H54A	0.5762	0.5119	0.5871	0.051*	
H54B	0.6173	0.3915	0.6155	0.051*	
C55	0.64248 (11)	0.6029 (4)	0.62611 (17)	0.0276 (8)	
H55A	0.6373	0.6046	0.5811	0.033*	
H55B	0.6349	0.7007	0.6344	0.033*	
C56	0.58360 (13)	0.5241 (4)	0.6999 (2)	0.0349 (9)	
C57	0.53659 (14)	0.5609 (4)	0.6434 (2)	0.0391 (10)	
H57	0.5279	0.5681	0.5979	0.047*	
C58	0.50404 (19)	0.5860 (5)	0.6558 (3)	0.0510 (14)	
H58	0.4721	0.6101	0.6184	0.061*	
C59	0.51660 (17)	0.5769 (4)	0.7216 (3)	0.0517 (12)	
H59	0.4937	0.5914	0.7302	0.062*	
C60	0.56340 (17)	0.5462 (4)	0.7745 (2)	0.0471 (11)	
H60	0.5727	0.5442	0.8204	0.057*	

### Atomic displacement parameters (Å^2^)

**Table:**

	*U*^11^	*U*^22^	*U*^33^	*U*^12^	*U*^13^	*U*^23^
Cd1	0.01918 (10)	0.02144 (11)	0.02824 (12)	0.00173 (13)	0.01579 (9)	0.00171 (14)
Cl2	0.0276 (4)	0.0357 (5)	0.0292 (5)	−0.0047 (4)	0.0161 (4)	−0.0045 (4)
Cl3	0.0321 (5)	0.0246 (4)	0.0327 (5)	−0.0055 (4)	0.0170 (4)	−0.0075 (4)
O1	0.070 (2)	0.122 (3)	0.083 (3)	−0.067 (2)	0.060 (2)	−0.063 (2)
O2	0.090 (3)	0.102 (3)	0.102 (3)	0.052 (3)	0.052 (3)	−0.015 (3)
O3	0.0525 (18)	0.0415 (17)	0.0557 (19)	−0.0073 (14)	0.0273 (16)	−0.0260 (15)
O4	0.087 (3)	0.141 (4)	0.047 (2)	−0.040 (3)	0.016 (2)	0.036 (2)
O5	0.068 (2)	0.0365 (17)	0.070 (2)	−0.0040 (16)	0.0344 (18)	−0.0245 (15)
O6	0.072 (2)	0.127 (3)	0.066 (2)	−0.015 (2)	0.054 (2)	−0.028 (2)
O7	0.054 (2)	0.095 (3)	0.069 (2)	−0.044 (2)	0.0321 (19)	−0.013 (2)
O8	0.099 (3)	0.058 (2)	0.046 (2)	0.024 (2)	0.013 (2)	0.0083 (18)
N1	0.0239 (15)	0.0237 (16)	0.0340 (17)	−0.0002 (13)	0.0185 (14)	−0.0007 (13)
N2	0.0174 (13)	0.0258 (15)	0.0247 (15)	0.0010 (12)	0.0108 (12)	0.0026 (13)
N3	0.0236 (16)	0.078 (3)	0.0260 (17)	−0.0031 (16)	0.0156 (14)	0.0052 (17)
N4	0.0385 (19)	0.048 (2)	0.0372 (19)	−0.0057 (16)	0.0275 (17)	−0.0030 (16)
N5	0.0341 (17)	0.0249 (16)	0.0371 (18)	−0.0026 (13)	0.0260 (15)	−0.0018 (14)
N6	0.0320 (16)	0.0282 (17)	0.0281 (16)	0.0009 (13)	0.0219 (14)	0.0022 (12)
N7	0.0299 (19)	0.160 (4)	0.029 (2)	0.020 (2)	0.0201 (17)	0.011 (2)
N8	0.0417 (19)	0.0376 (19)	0.041 (2)	0.0051 (15)	0.0270 (17)	−0.0016 (15)
N9	0.0259 (15)	0.0203 (15)	0.0351 (17)	0.0013 (12)	0.0218 (14)	0.0026 (13)
N10	0.0312 (16)	0.0288 (16)	0.0275 (16)	0.0042 (13)	0.0206 (14)	0.0046 (13)
N11	0.0300 (17)	0.068 (2)	0.0257 (17)	0.0036 (16)	0.0192 (15)	0.0062 (16)
N12	0.0437 (19)	0.0345 (19)	0.0314 (18)	0.0033 (16)	0.0280 (16)	0.0007 (15)
N13	0.0242 (15)	0.0233 (15)	0.0250 (15)	0.0018 (12)	0.0144 (13)	0.0013 (12)
N14	0.0195 (14)	0.0276 (16)	0.0215 (15)	0.0013 (11)	0.0120 (13)	0.0017 (12)
N15	0.0219 (17)	0.122 (4)	0.0266 (19)	−0.003 (2)	0.0131 (15)	0.021 (2)
N16	0.043 (2)	0.048 (2)	0.037 (2)	−0.0041 (17)	0.0266 (17)	0.0062 (17)
C1	0.0208 (17)	0.0207 (18)	0.036 (2)	−0.0029 (13)	0.0151 (16)	0.0011 (15)
C2	0.0287 (18)	0.0194 (19)	0.041 (2)	0.0009 (15)	0.0236 (17)	0.0016 (16)
C3	0.0248 (18)	0.0226 (19)	0.0264 (19)	0.0028 (15)	0.0159 (16)	0.0028 (15)
C4	0.0209 (19)	0.023 (2)	0.060 (3)	−0.0054 (16)	0.0160 (19)	−0.0059 (19)
C5	0.028 (2)	0.0152 (19)	0.067 (3)	0.0008 (17)	0.023 (2)	−0.0042 (19)
C6	0.023 (2)	0.0222 (18)	0.039 (2)	0.0034 (16)	0.0182 (19)	0.0045 (17)
C7	0.0215 (18)	0.046 (2)	0.028 (2)	0.0017 (17)	0.0130 (16)	−0.0028 (18)
C8	0.030 (2)	0.063 (3)	0.031 (2)	0.000 (2)	0.0166 (19)	0.009 (2)
C9	0.0205 (19)	0.063 (3)	0.036 (2)	−0.0066 (18)	0.0150 (18)	−0.001 (2)
C10	0.0239 (19)	0.046 (2)	0.028 (2)	0.0039 (16)	0.0142 (17)	0.0073 (17)
C11	0.033 (2)	0.035 (2)	0.032 (2)	−0.0071 (16)	0.0204 (18)	−0.0062 (17)
C12	0.034 (2)	0.033 (2)	0.037 (2)	−0.0074 (17)	0.0237 (19)	−0.0075 (17)
C13	0.034 (2)	0.030 (2)	0.057 (3)	−0.0085 (17)	0.029 (2)	−0.015 (2)
C14	0.052 (3)	0.031 (2)	0.057 (3)	−0.0052 (19)	0.044 (2)	−0.0086 (19)
C15	0.048 (3)	0.039 (2)	0.044 (2)	−0.0062 (19)	0.035 (2)	−0.0041 (19)
C16	0.060 (3)	0.024 (2)	0.061 (3)	−0.0109 (19)	0.052 (3)	−0.007 (2)
C17	0.056 (3)	0.026 (2)	0.062 (3)	−0.008 (2)	0.048 (2)	−0.012 (2)
C18	0.0266 (18)	0.0291 (19)	0.036 (2)	0.0028 (17)	0.0213 (16)	0.0065 (18)
C19	0.039 (2)	0.0249 (19)	0.047 (2)	0.0066 (17)	0.033 (2)	0.0072 (18)
C20	0.034 (2)	0.0236 (18)	0.040 (2)	0.0045 (15)	0.0264 (19)	0.0028 (16)
C21	0.051 (3)	0.031 (2)	0.051 (3)	0.0098 (19)	0.041 (2)	0.013 (2)
C22	0.033 (2)	0.061 (3)	0.028 (2)	−0.0069 (19)	0.0199 (18)	−0.0059 (19)
C23	0.037 (2)	0.126 (5)	0.030 (2)	0.025 (3)	0.023 (2)	0.015 (3)
C24	0.035 (2)	0.089 (4)	0.031 (2)	0.008 (2)	0.024 (2)	−0.005 (2)
C25	0.0303 (19)	0.048 (2)	0.0258 (19)	−0.0039 (18)	0.0186 (16)	−0.0014 (18)
C26	0.036 (2)	0.042 (2)	0.037 (2)	0.0125 (18)	0.024 (2)	0.0007 (19)
C27	0.041 (2)	0.054 (3)	0.035 (2)	0.008 (2)	0.024 (2)	0.000 (2)
C28	0.049 (3)	0.033 (2)	0.049 (3)	−0.0055 (18)	0.036 (2)	−0.0032 (18)
C29	0.038 (2)	0.025 (2)	0.053 (3)	−0.0047 (18)	0.024 (2)	−0.0035 (19)
C30	0.041 (2)	0.033 (2)	0.041 (3)	−0.0019 (18)	0.017 (2)	−0.0059 (18)
C31	0.044 (2)	0.027 (2)	0.050 (3)	0.0057 (18)	0.036 (2)	0.0023 (18)
C32	0.045 (2)	0.0186 (17)	0.052 (2)	0.0042 (18)	0.036 (2)	0.0075 (18)
C33	0.0322 (19)	0.030 (2)	0.030 (2)	0.0024 (17)	0.0207 (17)	0.0048 (17)
C34	0.044 (2)	0.026 (2)	0.039 (2)	0.0034 (18)	0.031 (2)	0.0035 (18)
C35	0.040 (3)	0.018 (2)	0.044 (3)	−0.0003 (16)	0.029 (2)	0.0013 (17)
C36	0.055 (3)	0.026 (2)	0.047 (3)	0.0066 (19)	0.039 (2)	0.0086 (19)
C37	0.042 (2)	0.051 (3)	0.028 (2)	−0.002 (2)	0.0251 (19)	0.0001 (19)
C38	0.036 (2)	0.077 (3)	0.024 (2)	0.006 (2)	0.0183 (18)	0.013 (2)
C39	0.036 (2)	0.052 (3)	0.023 (2)	0.0035 (18)	0.0198 (18)	0.0007 (18)
C40	0.036 (2)	0.036 (2)	0.025 (2)	0.0016 (17)	0.0195 (18)	0.0068 (16)
C41	0.038 (2)	0.035 (2)	0.027 (2)	0.0008 (17)	0.0218 (18)	−0.0016 (16)
C42	0.044 (2)	0.037 (2)	0.028 (2)	−0.0003 (18)	0.0226 (19)	0.0010 (17)
C43	0.049 (3)	0.022 (2)	0.042 (3)	−0.0039 (19)	0.035 (2)	−0.0027 (18)
C44	0.033 (2)	0.0233 (19)	0.050 (3)	−0.0039 (16)	0.024 (2)	−0.0056 (18)
C45	0.036 (2)	0.032 (2)	0.034 (2)	0.0004 (18)	0.0117 (19)	−0.0040 (18)
C46	0.0278 (19)	0.0271 (19)	0.037 (2)	−0.0060 (15)	0.0212 (17)	−0.0006 (16)
C47	0.0259 (18)	0.0238 (18)	0.032 (2)	−0.0006 (15)	0.0180 (16)	0.0037 (16)
C48	0.0247 (17)	0.0234 (17)	0.0207 (17)	−0.0015 (15)	0.0133 (15)	−0.0008 (15)
C49	0.0203 (17)	0.0209 (17)	0.0279 (19)	−0.0017 (14)	0.0127 (15)	−0.0006 (15)
C50	0.025 (2)	0.0175 (19)	0.032 (2)	−0.0021 (15)	0.0131 (17)	0.0007 (16)
C51	0.0193 (19)	0.023 (2)	0.031 (2)	0.0034 (15)	0.0128 (17)	0.0055 (17)
C52	0.0198 (17)	0.048 (3)	0.0270 (19)	0.0060 (16)	0.0109 (16)	0.0012 (17)
C53	0.027 (2)	0.085 (3)	0.025 (2)	−0.004 (2)	0.0127 (18)	0.016 (2)
C54	0.0239 (19)	0.072 (3)	0.027 (2)	−0.011 (2)	0.0122 (17)	0.006 (2)
C55	0.0207 (16)	0.037 (2)	0.0219 (17)	0.0029 (16)	0.0109 (14)	−0.0020 (16)
C56	0.036 (2)	0.035 (2)	0.037 (2)	−0.0055 (17)	0.0228 (19)	0.0108 (18)
C57	0.035 (2)	0.030 (2)	0.039 (2)	−0.0008 (17)	0.0152 (19)	0.0081 (17)
C58	0.037 (3)	0.032 (3)	0.074 (4)	0.010 (2)	0.028 (3)	0.001 (2)
C59	0.058 (3)	0.032 (3)	0.086 (4)	0.000 (2)	0.054 (3)	−0.008 (2)
C60	0.067 (3)	0.036 (2)	0.059 (3)	−0.007 (2)	0.048 (3)	−0.002 (2)

### Geometric parameters (Å, °)

**Table:**

Cd1—N13	2.310 (3)	C17—C18	1.377 (5)
Cd1—N1	2.320 (3)	C17—H17	0.9500
Cd1—N5	2.321 (3)	C18—C19	1.380 (5)
Cd1—N9	2.331 (3)	C18—C21	1.510 (5)
Cd1—O5	2.388 (3)	C19—C20	1.377 (5)
Cd1—O3	2.391 (3)	C19—H19	0.9500
Cl2—O4	1.394 (3)	C20—H20	0.9500
Cl2—O2	1.395 (3)	C21—H21A	0.9900
Cl2—O1	1.406 (3)	C21—H21B	0.9900
Cl2—O3	1.439 (3)	C22—C23	1.525 (6)
Cl3—O7	1.412 (3)	C22—H22A	0.9900
Cl3—O5	1.415 (3)	C22—H22B	0.9900
Cl3—O6	1.420 (3)	C23—H23A	0.9900
Cl3—O8	1.426 (3)	C23—H23B	0.9900
N1—C1	1.329 (4)	C24—C25	1.514 (5)
N1—C5	1.356 (5)	C24—H24A	0.9900
N2—C10	1.457 (4)	C24—H24B	0.9900
N2—C6	1.465 (5)	C25—H25A	0.9900
N2—C7	1.465 (4)	C25—H25B	0.9900
N3—C11	1.383 (4)	C26—C27	1.399 (5)
N3—C9	1.442 (5)	C27—C30	1.369 (6)
N3—C8	1.463 (5)	C27—H27	0.9500
N4—C11	1.333 (5)	C28—C29	1.367 (6)
N4—C15	1.346 (5)	C28—H28	0.9500
N5—C16	1.331 (5)	C29—C30	1.374 (6)
N5—C20	1.340 (4)	C29—H29	0.9500
N6—C21	1.459 (5)	C30—H30	0.9500
N6—C22	1.460 (5)	C31—C32	1.383 (5)
N6—C25	1.465 (4)	C31—H31	0.9500
N7—C26	1.366 (5)	C32—C33	1.380 (5)
N7—C23	1.446 (5)	C32—H32	0.9500
N7—C24	1.469 (5)	C33—C34	1.392 (5)
N8—C28	1.332 (5)	C33—C36	1.504 (5)
N8—C26	1.355 (5)	C34—C35	1.376 (6)
N9—C31	1.333 (5)	C34—H34	0.9500
N9—C35	1.336 (5)	C35—H35	0.9500
N10—C40	1.454 (4)	C36—H36A	0.9900
N10—C36	1.464 (5)	C36—H36B	0.9900
N10—C37	1.472 (4)	C37—C38	1.502 (5)
N11—C41	1.383 (4)	C37—H37A	0.9900
N11—C39	1.459 (5)	C37—H37B	0.9900
N11—C38	1.461 (5)	C38—H38A	0.9900
N12—C43	1.351 (5)	C38—H38B	0.9900
N12—C41	1.358 (4)	C39—C40	1.511 (5)
N13—C50	1.327 (5)	C39—H39A	0.9900
N13—C46	1.346 (4)	C39—H39B	0.9900
N14—C52	1.458 (4)	C40—H40A	0.9900
N14—C55	1.463 (4)	C40—H40B	0.9900
N14—C51	1.470 (5)	C41—C42	1.398 (5)
N15—C56	1.377 (5)	C42—C45	1.373 (5)
N15—C53	1.455 (5)	C42—H42	0.9500
N15—C54	1.455 (5)	C43—C44	1.374 (5)
N16—C56	1.342 (5)	C43—H43	0.9500
N16—C60	1.350 (5)	C44—C45	1.366 (5)
C1—C2	1.379 (5)	C44—H44	0.9500
C1—H1	0.9500	C45—H45	0.9500
C2—C3	1.388 (5)	C46—C47	1.386 (5)
C2—H2	0.9500	C46—H46	0.9500
C3—C4	1.377 (5)	C47—C48	1.379 (5)
C3—C6	1.502 (5)	C47—H47	0.9500
C4—C5	1.362 (6)	C48—C49	1.391 (5)
C4—H4	0.9500	C48—C51	1.510 (5)
C5—H5	0.9500	C49—C50	1.386 (5)
C6—H6A	0.9900	C49—H49	0.9500
C6—H6B	0.9900	C50—H50	0.9500
C7—C8	1.510 (5)	C51—H51A	0.9900
C7—H7A	0.9900	C51—H51B	0.9900
C7—H7B	0.9900	C52—C53	1.512 (5)
C8—H8A	0.9900	C52—H52A	0.9900
C8—H8B	0.9900	C52—H52B	0.9900
C9—C10	1.522 (5)	C53—H53A	0.9900
C9—H9A	0.9900	C53—H53B	0.9900
C9—H9B	0.9900	C54—C55	1.525 (5)
C10—H10A	0.9900	C54—H54A	0.9900
C10—H10B	0.9900	C54—H54B	0.9900
C11—C12	1.412 (5)	C55—H55A	0.9900
C12—C13	1.385 (5)	C55—H55B	0.9900
C12—H12	0.9500	C56—C57	1.409 (5)
C13—C14	1.370 (6)	C57—C58	1.359 (7)
C13—H13	0.9500	C57—H57	0.9500
C14—C15	1.366 (6)	C58—C59	1.374 (7)
C14—H14	0.9500	C58—H58	0.9500
C15—H15	0.9500	C59—C60	1.371 (6)
C16—C17	1.371 (6)	C59—H59	0.9500
C16—H16	0.9500	C60—H60	0.9500
			
N13—Cd1—N1	175.67 (11)	H22A—C22—H22B	108.2
N13—Cd1—N5	95.14 (10)	N7—C23—C22	110.5 (4)
N1—Cd1—N5	85.84 (10)	N7—C23—H23A	109.6
N13—Cd1—N9	86.92 (10)	C22—C23—H23A	109.6
N1—Cd1—N9	92.33 (10)	N7—C23—H23B	109.6
N5—Cd1—N9	176.34 (11)	C22—C23—H23B	109.6
N13—Cd1—O5	96.23 (10)	H23A—C23—H23B	108.1
N1—Cd1—O5	87.99 (11)	N7—C24—C25	110.1 (3)
N5—Cd1—O5	89.26 (11)	N7—C24—H24A	109.6
N9—Cd1—O5	87.51 (12)	C25—C24—H24A	109.6
N13—Cd1—O3	89.72 (10)	N7—C24—H24B	109.6
N1—Cd1—O3	85.96 (10)	C25—C24—H24B	109.6
N5—Cd1—O3	102.09 (11)	H24A—C24—H24B	108.2
N9—Cd1—O3	80.92 (10)	N6—C25—C24	110.1 (3)
O5—Cd1—O3	166.69 (12)	N6—C25—H25A	109.6
O4—Cl2—O2	108.8 (3)	C24—C25—H25A	109.6
O4—Cl2—O1	110.1 (2)	N6—C25—H25B	109.6
O2—Cl2—O1	110.7 (3)	C24—C25—H25B	109.6
O4—Cl2—O3	109.9 (2)	H25A—C25—H25B	108.2
O2—Cl2—O3	108.1 (2)	N8—C26—N7	116.9 (3)
O1—Cl2—O3	109.27 (19)	N8—C26—C27	120.6 (4)
O7—Cl3—O5	109.5 (2)	N7—C26—C27	122.5 (4)
O7—Cl3—O6	109.3 (2)	C30—C27—C26	119.2 (4)
O5—Cl3—O6	108.1 (2)	C30—C27—H27	120.4
O7—Cl3—O8	110.9 (2)	C26—C27—H27	120.4
O5—Cl3—O8	108.6 (2)	N8—C28—C29	124.9 (4)
O6—Cl3—O8	110.5 (3)	N8—C28—H28	117.6
Cl2—O3—Cd1	142.7 (2)	C29—C28—H28	117.6
Cl3—O5—Cd1	152.1 (2)	C28—C29—C30	117.1 (4)
C1—N1—C5	115.8 (3)	C28—C29—H29	121.5
C1—N1—Cd1	126.8 (2)	C30—C29—H29	121.5
C5—N1—Cd1	117.3 (2)	C27—C30—C29	120.4 (4)
C10—N2—C6	109.0 (3)	C27—C30—H30	119.8
C10—N2—C7	109.9 (3)	C29—C30—H30	119.8
C6—N2—C7	111.3 (3)	N9—C31—C32	123.2 (3)
C11—N3—C9	122.5 (3)	N9—C31—H31	118.4
C11—N3—C8	120.1 (3)	C32—C31—H31	118.4
C9—N3—C8	112.1 (3)	C33—C32—C31	120.0 (4)
C11—N4—C15	117.7 (3)	C33—C32—H32	120.0
C16—N5—C20	116.7 (3)	C31—C32—H32	120.0
C16—N5—Cd1	117.4 (3)	C32—C33—C34	117.0 (3)
C20—N5—Cd1	125.8 (2)	C32—C33—C36	120.8 (3)
C21—N6—C22	110.1 (3)	C34—C33—C36	122.2 (3)
C21—N6—C25	110.8 (3)	C35—C34—C33	119.2 (4)
C22—N6—C25	110.2 (3)	C35—C34—H34	120.4
C26—N7—C23	125.2 (3)	C33—C34—H34	120.4
C26—N7—C24	122.7 (3)	N9—C35—C34	124.0 (3)
C23—N7—C24	112.1 (3)	N9—C35—H35	118.0
C28—N8—C26	117.8 (4)	C34—C35—H35	118.0
C31—N9—C35	116.6 (3)	N10—C36—C33	112.7 (3)
C31—N9—Cd1	122.7 (2)	N10—C36—H36A	109.1
C35—N9—Cd1	120.0 (2)	C33—C36—H36A	109.1
C40—N10—C36	109.2 (3)	N10—C36—H36B	109.1
C40—N10—C37	108.4 (3)	C33—C36—H36B	109.1
C36—N10—C37	111.0 (3)	H36A—C36—H36B	107.8
C41—N11—C39	120.6 (3)	N10—C37—C38	110.6 (3)
C41—N11—C38	121.4 (3)	N10—C37—H37A	109.5
C39—N11—C38	112.7 (3)	C38—C37—H37A	109.5
C43—N12—C41	117.7 (3)	N10—C37—H37B	109.5
C50—N13—C46	116.6 (3)	C38—C37—H37B	109.5
C50—N13—Cd1	117.8 (2)	H37A—C37—H37B	108.1
C46—N13—Cd1	125.5 (2)	N11—C38—C37	109.8 (3)
C52—N14—C55	109.5 (3)	N11—C38—H38A	109.7
C52—N14—C51	111.6 (3)	C37—C38—H38A	109.7
C55—N14—C51	110.5 (3)	N11—C38—H38B	109.7
C56—N15—C53	122.4 (3)	C37—C38—H38B	109.7
C56—N15—C54	124.7 (3)	H38A—C38—H38B	108.2
C53—N15—C54	112.6 (3)	N11—C39—C40	110.2 (3)
C56—N16—C60	117.7 (4)	N11—C39—H39A	109.6
N1—C1—C2	123.6 (3)	C40—C39—H39A	109.6
N1—C1—H1	118.2	N11—C39—H39B	109.6
C2—C1—H1	118.2	C40—C39—H39B	109.6
C1—C2—C3	120.0 (3)	H39A—C39—H39B	108.1
C1—C2—H2	120.0	N10—C40—C39	111.9 (3)
C3—C2—H2	120.0	N10—C40—H40A	109.2
C4—C3—C2	116.7 (3)	C39—C40—H40A	109.2
C4—C3—C6	121.4 (3)	N10—C40—H40B	109.2
C2—C3—C6	121.8 (3)	C39—C40—H40B	109.2
C5—C4—C3	120.0 (3)	H40A—C40—H40B	107.9
C5—C4—H4	120.0	N12—C41—N11	116.7 (3)
C3—C4—H4	120.0	N12—C41—C42	121.5 (3)
N1—C5—C4	124.0 (4)	N11—C41—C42	121.7 (3)
N1—C5—H5	118.0	C45—C42—C41	118.5 (4)
C4—C5—H5	118.0	C45—C42—H42	120.8
N2—C6—C3	113.3 (3)	C41—C42—H42	120.8
N2—C6—H6A	108.9	N12—C43—C44	123.4 (4)
C3—C6—H6A	108.9	N12—C43—H43	118.3
N2—C6—H6B	108.9	C44—C43—H43	118.3
C3—C6—H6B	108.9	C45—C44—C43	118.2 (4)
H6A—C6—H6B	107.7	C45—C44—H44	120.9
N2—C7—C8	110.8 (3)	C43—C44—H44	120.9
N2—C7—H7A	109.5	C44—C45—C42	120.7 (4)
C8—C7—H7A	109.5	C44—C45—H45	119.6
N2—C7—H7B	109.5	C42—C45—H45	119.6
C8—C7—H7B	109.5	N13—C46—C47	123.0 (3)
H7A—C7—H7B	108.1	N13—C46—H46	118.5
N3—C8—C7	110.0 (3)	C47—C46—H46	118.5
N3—C8—H8A	109.7	C48—C47—C46	120.0 (3)
C7—C8—H8A	109.7	C48—C47—H47	120.0
N3—C8—H8B	109.7	C46—C47—H47	120.0
C7—C8—H8B	109.7	C47—C48—C49	117.3 (3)
H8A—C8—H8B	108.2	C47—C48—C51	120.2 (3)
N3—C9—C10	110.5 (3)	C49—C48—C51	122.5 (3)
N3—C9—H9A	109.6	C50—C49—C48	119.0 (3)
C10—C9—H9A	109.6	C50—C49—H49	120.5
N3—C9—H9B	109.6	C48—C49—H49	120.5
C10—C9—H9B	109.6	N13—C50—C49	124.1 (3)
H9A—C9—H9B	108.1	N13—C50—H50	117.9
N2—C10—C9	110.8 (3)	C49—C50—H50	117.9
N2—C10—H10A	109.5	N14—C51—C48	112.8 (3)
C9—C10—H10A	109.5	N14—C51—H51A	109.0
N2—C10—H10B	109.5	C48—C51—H51A	109.0
C9—C10—H10B	109.5	N14—C51—H51B	109.0
H10A—C10—H10B	108.1	C48—C51—H51B	109.0
N4—C11—N3	116.2 (3)	H51A—C51—H51B	107.8
N4—C11—C12	122.3 (3)	N14—C52—C53	110.9 (3)
N3—C11—C12	121.4 (3)	N14—C52—H52A	109.5
C13—C12—C11	117.3 (4)	C53—C52—H52A	109.5
C13—C12—H12	121.3	N14—C52—H52B	109.5
C11—C12—H12	121.3	C53—C52—H52B	109.5
C14—C13—C12	120.7 (4)	H52A—C52—H52B	108.0
C14—C13—H13	119.7	N15—C53—C52	110.8 (3)
C12—C13—H13	119.7	N15—C53—H53A	109.5
C15—C14—C13	117.8 (4)	C52—C53—H53A	109.5
C15—C14—H14	121.1	N15—C53—H53B	109.5
C13—C14—H14	121.1	C52—C53—H53B	109.5
N4—C15—C14	124.1 (4)	H53A—C53—H53B	108.1
N4—C15—H15	117.9	N15—C54—C55	109.5 (3)
C14—C15—H15	117.9	N15—C54—H54A	109.8
N5—C16—C17	123.6 (4)	C55—C54—H54A	109.8
N5—C16—H16	118.2	N15—C54—H54B	109.8
C17—C16—H16	118.2	C55—C54—H54B	109.8
C16—C17—C18	120.0 (4)	H54A—C54—H54B	108.2
C16—C17—H17	120.0	N14—C55—C54	108.8 (3)
C18—C17—H17	120.0	N14—C55—H55A	109.9
C17—C18—C19	116.7 (3)	C54—C55—H55A	109.9
C17—C18—C21	121.8 (3)	N14—C55—H55B	109.9
C19—C18—C21	121.6 (3)	C54—C55—H55B	109.9
C20—C19—C18	120.3 (3)	H55A—C55—H55B	108.3
C20—C19—H19	119.8	N16—C56—N15	116.7 (3)
C18—C19—H19	119.8	N16—C56—C57	121.8 (4)
N5—C20—C19	122.7 (3)	N15—C56—C57	121.5 (4)
N5—C20—H20	118.7	C58—C57—C56	118.1 (4)
C19—C20—H20	118.7	C58—C57—H57	120.9
N6—C21—C18	112.7 (3)	C56—C57—H57	120.9
N6—C21—H21A	109.1	C57—C58—C59	121.1 (5)
C18—C21—H21A	109.1	C57—C58—H58	119.5
N6—C21—H21B	109.1	C59—C58—H58	119.5
C18—C21—H21B	109.1	C60—C59—C58	117.6 (4)
H21A—C21—H21B	107.8	C60—C59—H59	121.2
N6—C22—C23	110.0 (3)	C58—C59—H59	121.2
N6—C22—H22A	109.7	N16—C60—C59	123.6 (4)
C23—C22—H22A	109.7	N16—C60—H60	118.2
N6—C22—H22B	109.7	C59—C60—H60	118.2
C23—C22—H22B	109.7		
			
O4—Cl2—O3—Cd1	−17.1 (4)	C19—C18—C21—N6	150.0 (4)
O1—Cl2—O3—Cd1	−137.9 (3)	C21—N6—C22—C23	178.3 (3)
N13—Cd1—O3—Cl2	87.4 (3)	C25—N6—C22—C23	−59.1 (4)
N1—Cd1—O3—Cl2	−92.7 (3)	C26—N7—C23—C22	124.2 (5)
N5—Cd1—O3—Cl2	−7.8 (3)	C24—N7—C23—C22	−55.7 (6)
N9—Cd1—O3—Cl2	174.3 (3)	N6—C22—C23—N7	56.9 (5)
O5—Cd1—O3—Cl2	−155.8 (4)	C26—N7—C24—C25	−124.0 (5)
O7—Cl3—O5—Cd1	−149.1 (5)	C23—N7—C24—C25	55.9 (6)
O6—Cl3—O5—Cd1	92.0 (5)	C21—N6—C25—C24	−178.3 (3)
O8—Cl3—O5—Cd1	−27.9 (6)	C22—N6—C25—C24	59.5 (4)
N13—Cd1—O5—Cl3	−11.3 (5)	N7—C24—C25—N6	−57.0 (5)
N1—Cd1—O5—Cl3	169.6 (5)	C28—N8—C26—N7	−177.9 (4)
N5—Cd1—O5—Cl3	83.7 (5)	C28—N8—C26—C27	0.7 (6)
N9—Cd1—O5—Cl3	−98.0 (5)	C23—N7—C26—N8	178.3 (5)
O3—Cd1—O5—Cl3	−127.5 (5)	C24—N7—C26—N8	−1.8 (7)
N5—Cd1—N1—C1	−116.7 (3)	C23—N7—C26—C27	−0.3 (8)
N9—Cd1—N1—C1	66.5 (3)	C24—N7—C26—C27	179.6 (4)
O5—Cd1—N1—C1	153.9 (3)	N8—C26—C27—C30	−0.1 (6)
O3—Cd1—N1—C1	−14.2 (3)	N7—C26—C27—C30	178.5 (4)
N5—Cd1—N1—C5	59.7 (3)	C26—N8—C28—C29	−0.5 (6)
N9—Cd1—N1—C5	−117.1 (3)	N8—C28—C29—C30	−0.4 (6)
O5—Cd1—N1—C5	−29.7 (3)	C26—C27—C30—C29	−0.9 (6)
O3—Cd1—N1—C5	162.1 (3)	C28—C29—C30—C27	1.1 (6)
N13—Cd1—N5—C16	−123.3 (3)	C35—N9—C31—C32	0.1 (6)
N1—Cd1—N5—C16	52.5 (3)	Cd1—N9—C31—C32	−170.4 (3)
O5—Cd1—N5—C16	140.5 (3)	N9—C31—C32—C33	0.8 (6)
O3—Cd1—N5—C16	−32.5 (3)	C31—C32—C33—C34	−1.4 (6)
N13—Cd1—N5—C20	60.9 (3)	C31—C32—C33—C36	−179.7 (4)
N1—Cd1—N5—C20	−123.4 (3)	C32—C33—C34—C35	1.0 (6)
O5—Cd1—N5—C20	−35.3 (3)	C36—C33—C34—C35	179.4 (4)
O3—Cd1—N5—C20	151.7 (3)	C31—N9—C35—C34	−0.5 (6)
N13—Cd1—N9—C31	123.1 (3)	Cd1—N9—C35—C34	170.3 (3)
N1—Cd1—N9—C31	−52.6 (3)	C33—C34—C35—N9	−0.1 (6)
O5—Cd1—N9—C31	−140.5 (3)	C40—N10—C36—C33	−173.2 (3)
O3—Cd1—N9—C31	32.9 (3)	C37—N10—C36—C33	67.4 (4)
N13—Cd1—N9—C35	−47.1 (3)	C32—C33—C36—N10	−148.3 (4)
N1—Cd1—N9—C35	137.1 (3)	C34—C33—C36—N10	33.4 (6)
O5—Cd1—N9—C35	49.3 (3)	C40—N10—C37—C38	60.5 (4)
O3—Cd1—N9—C35	−137.3 (3)	C36—N10—C37—C38	−179.6 (3)
N5—Cd1—N13—C50	134.0 (3)	C41—N11—C38—C37	−150.2 (4)
N9—Cd1—N13—C50	−49.1 (3)	C39—N11—C38—C37	55.3 (5)
O5—Cd1—N13—C50	−136.2 (3)	N10—C37—C38—N11	−58.7 (4)
O3—Cd1—N13—C50	31.9 (3)	C41—N11—C39—C40	152.0 (4)
N5—Cd1—N13—C46	−49.2 (3)	C38—N11—C39—C40	−53.3 (4)
N9—Cd1—N13—C46	127.7 (3)	C36—N10—C40—C39	−180.0 (3)
O5—Cd1—N13—C46	40.6 (3)	C37—N10—C40—C39	−59.0 (4)
O3—Cd1—N13—C46	−151.3 (3)	N11—C39—C40—N10	55.6 (4)
C5—N1—C1—C2	0.1 (6)	C43—N12—C41—N11	−175.4 (3)
Cd1—N1—C1—C2	176.5 (3)	C43—N12—C41—C42	2.6 (5)
N1—C1—C2—C3	−0.2 (6)	C39—N11—C41—N12	−21.3 (5)
C1—C2—C3—C4	−0.2 (5)	C38—N11—C41—N12	−173.8 (4)
C1—C2—C3—C6	177.3 (3)	C39—N11—C41—C42	160.7 (4)
C2—C3—C4—C5	0.6 (6)	C38—N11—C41—C42	8.3 (6)
C6—C3—C4—C5	−176.8 (4)	N12—C41—C42—C45	−2.3 (6)
C1—N1—C5—C4	0.4 (7)	N11—C41—C42—C45	175.6 (4)
Cd1—N1—C5—C4	−176.4 (4)	C41—N12—C43—C44	−2.0 (6)
C3—C4—C5—N1	−0.8 (7)	N12—C43—C44—C45	1.1 (6)
C10—N2—C6—C3	168.5 (3)	C43—C44—C45—C42	−0.8 (6)
C7—N2—C6—C3	−70.1 (4)	C41—C42—C45—C44	1.4 (6)
C4—C3—C6—N2	−36.9 (5)	C50—N13—C46—C47	0.3 (5)
C2—C3—C6—N2	145.8 (3)	Cd1—N13—C46—C47	−176.5 (3)
C10—N2—C7—C8	−58.2 (4)	N13—C46—C47—C48	0.0 (5)
C6—N2—C7—C8	−179.1 (3)	C46—C47—C48—C49	−0.6 (5)
C11—N3—C8—C7	148.9 (4)	C46—C47—C48—C51	−177.6 (3)
C9—N3—C8—C7	−56.2 (5)	C47—C48—C49—C50	0.8 (5)
N2—C7—C8—N3	56.9 (4)	C51—C48—C49—C50	177.8 (4)
C11—N3—C9—C10	−150.0 (4)	C46—N13—C50—C49	0.0 (6)
C8—N3—C9—C10	55.8 (5)	Cd1—N13—C50—C49	177.0 (3)
C6—N2—C10—C9	179.8 (3)	C48—C49—C50—N13	−0.5 (6)
C7—N2—C10—C9	57.6 (4)	C52—N14—C51—C48	76.1 (4)
N3—C9—C10—N2	−56.5 (4)	C55—N14—C51—C48	−161.9 (3)
C15—N4—C11—N3	−176.1 (3)	C47—C48—C51—N14	−166.4 (3)
C15—N4—C11—C12	1.4 (6)	C49—C48—C51—N14	16.7 (5)
C9—N3—C11—N4	−164.0 (4)	C55—N14—C52—C53	59.5 (4)
C8—N3—C11—N4	−11.8 (5)	C51—N14—C52—C53	−177.9 (3)
C9—N3—C11—C12	18.4 (6)	C56—N15—C53—C52	−119.4 (4)
C8—N3—C11—C12	170.6 (4)	C54—N15—C53—C52	53.7 (5)
N4—C11—C12—C13	−1.4 (6)	N14—C52—C53—N15	−54.6 (5)
N3—C11—C12—C13	176.0 (4)	C56—N15—C54—C55	116.4 (4)
C11—C12—C13—C14	1.2 (6)	C53—N15—C54—C55	−56.5 (5)
C12—C13—C14—C15	−1.1 (6)	C52—N14—C55—C54	−61.8 (4)
C11—N4—C15—C14	−1.3 (6)	C51—N14—C55—C54	174.9 (3)
C13—C14—C15—N4	1.1 (6)	N15—C54—C55—N14	60.0 (4)
C20—N5—C16—C17	0.5 (7)	C60—N16—C56—N15	−176.7 (4)
Cd1—N5—C16—C17	−175.7 (4)	C60—N16—C56—C57	1.6 (6)
N5—C16—C17—C18	−0.3 (8)	C53—N15—C56—N16	−7.2 (6)
C16—C17—C18—C19	−0.3 (6)	C54—N15—C56—N16	−179.5 (4)
C16—C17—C18—C21	−179.9 (4)	C53—N15—C56—C57	174.5 (4)
C17—C18—C19—C20	0.6 (5)	C54—N15—C56—C57	2.2 (7)
C21—C18—C19—C20	−179.8 (4)	N16—C56—C57—C58	−2.4 (6)
C16—N5—C20—C19	−0.1 (5)	N15—C56—C57—C58	175.8 (4)
Cd1—N5—C20—C19	175.7 (3)	C56—C57—C58—C59	0.5 (6)
C18—C19—C20—N5	−0.4 (6)	C57—C58—C59—C60	2.0 (6)
C22—N6—C21—C18	−72.6 (4)	C56—N16—C60—C59	1.1 (6)
C25—N6—C21—C18	165.2 (3)	C58—C59—C60—N16	−2.9 (6)
C17—C18—C21—N6	−30.5 (5)		

### Hydrogen-bond geometry (Å, °)

**Table:**

*D*—H···*A*	*D*—H	H···*A*	*D*···*A*	*D*—H···*A*
C29—H29···O1^i^	0.95	2.54	3.406 (6)	152
C54—H54A···O7^ii^	0.99	2.57	3.348 (5)	135

Symmetry codes: (i) *x*+1/2, *y*+1/2, *z*; (ii) *x*−1/2, −*y*+3/2, *z*−1/2.
